# A two-phase case–control study for colorectal cancer genetic susceptibility: candidate genes from chromosomal regions 9q22 and 3q22

**DOI:** 10.1038/bjc.2011.296

**Published:** 2011-08-02

**Authors:** A Abulí, C Fernández-Rozadilla, M D Giráldez, J Muñoz, V Gonzalo, X Bessa, L Bujanda, J M Reñé, A Lanas, A M García, J Saló, L Argüello, À Vilella, R Carreño, R Jover, R M Xicola, X Llor, L Carvajal-Carmona, I P M Tomlinson, D J Kerr, R S Houlston, J M Piqué, A Carracedo, A Castells, M Andreu, C Ruiz-Ponte, S Castellví-Bel

**Affiliations:** 1 Department of Gastroenterology, Hospital Clínic, CIBERehd, IDIBAPS, University of Barcelona, Villarroel 170, 08036, Barcelona, Catalonia, Spain; 2 Gastroenterology Department, Parc de Salut Mar, Institut Municipal d’Investigació Mèdica (IMIM), Pompeu Fabra University, Barcelona, Catalonia, Spain; 3 Galician Public Foundation of Genomic Medicine (FPGMX), CIBERER, Genomics Medicine Group, Hospital Clínico, Santiago de Compostela, University of Santiago de Compostela, Galicia, Spain; 4 Department of Gastroenterology, Hospital de Donostia, CIBERehd, University of Basque Country, San Sebastian, Spain; 5 Department of Gastroenterology, Hospital Universitari Arnau de Vilanova, Lleida, Catalonia, Spain; 6 Department of Gastroenterology, Hospital Clínico Universitario, CIBERehd, Zaragoza, Zaragoza, Spain; 7 Fundación para la Formación e Investigación Sanitaria, Murcia, Spain; 8 Department of Gastroenterology, Hospital General de Vic, Barcelona, Catalonia, Spain; 9 Department of Gastroenterology, Hospital Universitario La Fe, Valencia, Valencia, Spain; 10 Department of Medicine, Hospital Son Llatzer, Palma de Mallorca, Balearic Islands, Spain; 11 Department of Gastroenterology, Fundación Hospitalaria de Calahorra, Navarre, Spain; 12 Department of Gastroenterology, Hospital General d’Alacant, Alicante, Spain; 13 Section of Digestive Diseases and Nutrition, University of Illinois at Chicago, Chicago, IL, USA; 14 Wellcome Trust Centre for Human Genetics, University of Oxford, Oxford, UK; 15 Department of Clinical Pharmacology, University of Oxford, Oxford, UK; 16 Section of Cancer Genetics, Institute of Cancer Research, Sutton, UK

**Keywords:** colorectal neoplasm, genetic predisposition to disease, single-nucleotide polymorphism, genetic association study

## Abstract

**Background:**

Colorectal cancer (CRC) is the second cause of cancer-related death in the Western world. Much of the CRC genetic risk remains unidentified and may be attributable to a large number of common, low-penetrance genetic variants. Genetic linkage studies in CRC families have reported additional association with regions 9q22–31, 3q21–24, 7q31, 11q, 14q and 22q. There are several plausible candidate genes for CRC susceptibility within the aforementioned linkage regions including *PTCH1*, *XPA* and *TGFBR1* in 9q22–31, and *EPHB1* and *MRAS* in 3q21–q24.

**Methods:**

CRC cases and matched controls were from EPICOLON, a prospective, multicentre, nationwide Spanish initiative, composed of two independent phases. Phase 1 corresponded to 515 CRC cases and 515 controls, whereas phase 2 consisted of 901 CRC cases and 909 controls. Genotyping was performed for 172 single-nucleotide polymorphisms (SNPs) in 84 genes located within regions 9q22–31 and 3q21–q24.

**Results:**

None of the 172 SNPs analysed in our study could be formally associated with CRC risk. However, rs1444601 (*TOPBP1*) and rs13088006 (*CDV3*) in region 3q22 showed interesting results and may have an effect on CRC risk.

**Conclusions:**

*TOPBP1* and *CDV3* genetic variants on region 3q22 may modulate CRC risk. Further validation and meta-analysis should be undertaken in larger CRC cohorts.

Colorectal cancer (CRC) continues to be a major public health problem, although it is a preventable and potentially curable neoplasm. Colorectal cancer is the second most common cancer in Western countries and it also represents the second leading cause of cancer-related death among men and women (Ferlay *et al*, 2010). Genetic and environmental factors are important in its pathogenesis. Although the majority of CRC is sporadic, inherited susceptibility is relevant in about 30–35% of cases. Germline mutations in known genes such as *APC* and the DNA mismatch repair family account for <6% of cases (Piñol *et al*, 2005; Jasperson *et al*, 2010). Much of the remaining genetic risk may be attributable to a large number of common, low-penetrance genetic variants each exerting a small influence on risk and following a polygenic model of inheritance (Balmain *et al*, 2003).

Genetic association studies are among the possible approaches to identify genes that underlie common diseases, either by candidate gene selection or GWAS. Recent GWASs have robustly demonstrated that common genetic variation contributes to the risk of developing CRC, and an increasing number of genomic regions have been shown to be associated with CRC risk. So far, 14 common, low-penetrant genetic variants have been identified for CRC susceptibility on 8q24.21, 18q21.1, 15q13.3, 8q23.3, 10p14, 11q23.1, 14q22.2, 16q22.1, 19q13, 20p12.3, 1q41, 3q26.2, 12q13.13 and 20q13.33 (Tenesa and Dunlop, 2009; Houlston *et al*, 2010). Previously, genetic linkage studies in CRC families additionally reported association with regions 9q22–31 (Wiesner *et al*, 2003; Skoglund *et al*, 2006; Kemp *et al*, 2006a; Gray-McGuire *et al*, 2010), 3q21–24 (Kemp *et al*, 2006b; Papaemmanuil *et al*, 2008; Picelli *et al*, 2008; Middeldorp *et al*, 2010), 7q31 (Neklason *et al*, 2008), 11q, 14q and 22q (Djureinovic *et al*, 2006). Wiesner *et al* (2003) reported genetic linkage to chromosomal region 9q22.2–31.2 in a set of 74 affected sibling pairs from 53 kindred with multiple CRC and/or advanced colorectal adenoma cases. Subsequently, Skoglund *et al* (2006) confirmed, in one extended family, a linkage region on 9q22.32–31.1 associated with adenoma and CRC predisposition. In addition, Kemp *et al* (2006a) suggested further evidence of a CRC susceptibility locus on 9q22.32–q31.1 in 57 CRC families from the United Kingdom. Recently, Gray-McGuire *et al* (2010) refined CRC linkage on 9q22–31 and narrowed it down to a 151-kb region using an additional cohort of 69 independent CRC kindred. On the other hand, Kemp *et al* (2006b) provided evidence of the existence of a novel CRC predisposition region on chromosome 3q21–q24 through a genome-wide linkage analysis in 69 CRC families, which was subsequently extended by Papaemmanuil *et al* (2008) in 34 additional CRC families. Two other independent linkage studies have also pointed out a region on chromosome 3q21–q24 to be linked to CRC susceptibility (Picelli *et al*, 2008; Middeldorp *et al*, 2010).

There are several plausible candidate genes for CRC susceptibility within the aforementioned linkage regions, including *PTCH1*, *XPA* and *TGFBR1* in 9q22–31, and *EPHB1* and *MRAS* in 3q21–q24. However, variations within these genes or other interesting candidate genes have been hardly evaluated in case–control genetic association studies to assess them as CRC genetic susceptibility components.

Hence, the aim of our study was to select candidate genes/variants from 9q22 and 3q22 and to consider their potential implications in CRC genetic susceptibility. Thus, we performed a two-phase case–control association study in the EPICOLON cohort (1416 CRC cases and 1424 controls) analysing 172 single-nucleotide polymorphisms (SNPs) within 84 genes located within these chromosomal regions.

## Patients and methods

### Study subjects

We included 1416 CRC patients and 1424 controls from the Spanish population. Cases and healthy controls were recruited in the EPICOLON project, a prospective, multicentre, population-based cohort, in two independent phases (2000–2001 and 2006–2008). The EPICOLON cohort has been described in detail elsewhere (Piñol *et al*, 2005; Abulí *et al*, 2010). The mean age at CRC diagnosis was 70 years, early-onset CRC (<50) was present at a 4–5% frequency and ∼15% of cases has a first-degree relative with CRC. Subjects in the discovery phase (phase 1) included 515 CRC cases and 515 controls. Subjects in the replication phase (phase 2) comprised 901 CRC cases and 909 controls. Exclusion criteria for the case–control study were hereditary CRC forms (familial adenomatous polyposis, MUTYH-associated polyposis and Lynch's syndrome) and personal history of inflammatory bowel disease. Cases and controls were gender and age matched (±5 years) and controls lacked personal and family cancer history. DNA samples were extracted as described previously (Castellví-Bel *et al*, 2007; Fernández-Rozadilla *et al*, 2010). This study was approved by the institutional ethics committees of each participating hospital and written informed consent was obtained from all individuals.

### Candidate gene selection

Linkage region on chromosome 9 was delimited between 90 795 373 and 106 903 700 bp, spanning 16.1 Mb, whereas linkage region on chromosome 3 was contained between 129 274 056 and 140 286 919 bp covering 11.01 Mb (according to NCBI genome build 37.2, http://www.ncbi.nlm.nih.gov). Gene selection was biased to include genes with previous evidence of being involved in cancer (e.g., DNA repair genes), with a function compatible with cancer involvement (e.g., cell cycle) or with a gene ontology term suggestive of a role in cancer (e.g., DNA binding). Pseudogenes were excluded from gene selection. In all, 41 out of 207 genes and 43 out of 123 genes were selected within the delimited regions on chromosomes 9 and 3, respectively, adding up to a total of 84. Description of all selected genes is summarised in Supplementary Tables 1 and 2. It is worth mentioning that gene selection was limited as it was based on current gene function annotations on available databases. Nowadays, it is estimated that gene function is known for 10–30% of genes in the human genome.

### SNP selection and genotyping

Overall, 94 SNPs from 41 genes and 78 SNPs from 43 genes were selected in regions 9q22 and 3q22, respectively, adding up to 172 variants. Single-nucleotide polymorphisms were chosen using only a direct strategy, selecting variants within each gene with a putative functional effect by using PupaSuite, a web tool used for the selection of genetic variants with potential phenotypic effect (Conde *et al*, 2006; http://pupasuite.bioinfo.cipf.es). TagSNPs were not selected in our study and segregation in the same haplotype block was not considered for exclusion. Single-nucleotide polymorphisms were always prioritised if they were coding, evolutionary conserved in mouse, with a putative regulatory effect in promoter, intronic or 3′-UTR regions, or involved in microRNAs binding. Minor allele frequency (MAF) was always >5%. One or two SNPs were usually selected from each gene, although more SNPs per gene were included if gene functionality was considered more important for CRC susceptibility. A description of all selected SNPs from regions 9q22 and 3q22 is available in Supplementary Tables 1 and 2, respectively.

High-throughput genotyping was performed according to the manufacturer's instructions using the SNPlex system (Applied Biosystems, Foster City, CA, USA), or the single-base primer extension chemistry matrix-assisted laser desorption/ionisation time-of-flight mass spectrometry detection platform (Sequenom Inc., San Diego, CA, USA). For rs11466445 (*TGFBR1*), a specific PCR followed by single-strand conformation polymorphism detection was performed to identify a 9-bp allele difference (deletion of GGCGGCGGC). This SNP was not possible to genotype with high-throughput technology and it was only genotyped in EPICOLON 1 samples. Genotyping was performed at the Santiago de Compostela and Barcelona nodes of the Spanish National Genotyping Centre (www.cegen.org).

### Statistical analysis

Genotyping quality control in both cohorts was performed using PLINK v1.03 (Purcell *et al*, 2007; http://pngu.mgh.harvard.edu/purcell/plink) excluding SNPs with genotype success rates below 90% and individuals with genotype success rates below 80%. Departure from the Hardy–Weinberg equilibrium for all biallelic SNP markers was tested in controls using a *χ*^2^-test with a single degree of freedom to exclude genotyping artefacts. After quality control, 991 samples (487 cases and 504 controls) and 170 SNPs remained to be analysed on phase 1. On the other hand, 1685 samples (847 cases and 838 controls) and 20 SNPs remained to be analysed on phase 2. There was no sign of underlying population stratification in EPICOLON as tested by an independent study (Fernández-Rozadilla *et al*, 2010). Genotypic/allelic association tests and logistic regression analyses were performed using PLINK v1.03. Genotype frequency differences were evaluated by regression analysis for allelic, genotypic, dominant and recessive models of inheritance. We estimated the crude odds ratio (OR) and 95% confidence intervals. If one of the genotypes had a frequency <5, then Fisher's exact test was used. In EPICOLON phase 1, a liberal *P*-value threshold (*P*-value <0.05) was used to avoid false-negative results. We then validated statistically significant phase 1 results by replicating them in another independent CRC cohort (EPICOLON phase 2). Although they were not statistically significant in EPICOLON phase 1, rs1800975 (*XPA*) and rs357564 (*PTCH1*) were also moved forward to phase 2 because of their biological interest. Finally, to address the issue of multiple testing, we used Bonferroni's correction (*P*=0.0025 for 20 SNPs). Study power was estimated using CATS software (Skol *et al*, 2006). Association results in EPICOLON for some SNPs were also evaluated in two cohorts described in a previous GWAS (Tomlinson *et al*, 2007), either by checking the original variant or a proxy SNP highly correlated with it (*r*^2^>0.7). These cohorts were CORGI (1432 CRC cases and 2697 controls) and VQ58 (928 CRC cases and 931 controls).

## Results

After genotyping quality control, data from 170 SNPs in 84 genes were available in EPICOLON phase 1 and all SNPs analysed were in the Hardy–Weinberg equilibrium in controls (*P*-value >0. 01). After association analysis in EPICOLON phase 1, 21 SNPs were statistically significant with an unadjusted *P*-value <0.05 in any of the tested inheritance models (Table 1). It is worth mentioning that eight of the significant SNPs in phase 1 were located in region 9q22, three of them in the *ABCA1* gene. On the other hand, the remaining 13 significant SNPs were located in region 3q22, 2 SNPs each in the *A4GNT* and *ARMC8* genes.

In the EPICOLON phase 1 cohort, a liberal *P*-value threshold (*P*-value <0.05) was used to avoid false-negative results. We then validated statistically significant phase 1 results by replicating them in another independent CRC cohort (EPICOLON phase 2). Two SNPs with non-statistically significant results in phase 1 from relevant genes in 9q22 (rs357564 in *PTCH1* and rs1800975 in *XPA*) were pushed forward to phase 2 to achieve results in both cohorts. In addition, rs11466445 in *TGFBR1*, although being borderline significant in EPICOLON phase 1, was not genotyped in phase 2 because of incompatibility with the used high-throughput technology. Genotype frequencies of all variants in the EPICOLON phase 2 control population fitted the Hardy–Weinberg equilibrium (*P*>0.01), except for rs12236219 and rs3738000 that were excluded. Therefore, results were available in EPICOLON phases 1 and 2 for the remaining 20 SNPs. For them, we performed a joint analysis of data combining both cohorts (1361 CRC cases and 1342 controls) to improve statistical power, as suggested previously (Skol *et al*, 2006).

Taking into account both EPICOLON phases, six SNPs in region 3q22 (rs1444601, rs13088006, rs939453, rs1131597, rs10934954 and rs2071387) maintained statistical significance with unadjusted *P*-value in the overall analysis. Association analysis for these six SNPs in the discovery, replication and overall cohorts is shown in Table 2. Among them, the most interesting results were achieved by rs1444601 in *TOPBP1* and rs13088006 in *CDV3*, maintaining statistical significance with an unadjusted *P*-value in all phases. However, it is worth mentioning that these observed associations would not be present if Bonferroni's correction for multiple testing was applied (*P*=0.0025 for 20 SNPs) and, therefore, they should be considered formally as not statistically significant.

In addition, association results for rs1444601, rs13088006, rs939453, rs1131597, rs10934954 and rs2071387 were also evaluated in two cohorts described in a previous GWAS (Tomlinson *et al*, 2007), either by checking the original variant or a proxy SNP highly correlated with it (*r*^2^>0.7) (Table 3). None of these six variants were significantly associated with CRC risk in this previous GWAS.

## Discussion

Several genetic linkage studies in CRC families have previously reported disease association with chromosomal regions 9q22–31 and 3q21–24. There are several plausible candidate genes for CRC susceptibility within the aforementioned linkage regions including *PTCH1*, *XPA* and *TGFBR1* in 9q22–31, and *EPHB1* and *MRAS* in 3q21–q24. Therefore, our study selected candidate genes/variants from 9q22 and 3q22 and evaluated its potential implication in CRC genetic susceptibility. For these means, we performed a two-phase case–control association study in the EPICOLON cohort (1416 CRC cases and 1424 controls) analysing 172 SNPs within 84 genes located within chromosomal regions 9q22–31 and 3q21–q24 potentially involved in cancer. For instance, among the selected genes were phosphatidylinositol 3-kinases, a gene family involved in multiple cellular functions related to cancer, or *MRAS*, part of the Ras signalling extensively dysregulated in carcinogenesis (Bunney and Katan, 2010).

The *TGFBR1* gene located in chromosomal region 9q22 was also incorporated in our study, and SNPs within this gene included rs11466445, a polymorphic 9-bp deletion with controversial results regarding its association with CRC risk (Kaklamani *et al*, 2003; Skoglund *et al*, 2007). Our results in the first phase showed a borderline significant association (unadjusted *P*-value =0.0491, dominant inheritance) but did not reach statistical significance after multiple-testing correction, suggesting that rs11466445 does not increase CRC risk. We also screened six other TGFBR1 variants with potential pathogenic effects and found no evidence of CRC risk association, in agreement with recent studies in Spanish and northern European populations (Castillejo *et al*, 2009; Carvajal-Carmona *et al*, 2010). Besides, the transforming growth factor-*β* (TGF-*β*) pathway has been strongly involved in CRC carcinogenesis, and its signalling is dependent on both receptors, TGFBR1 and TGFBR2. Although mutations in the *TGFBR2* gene have been explicitly associated with CRC, the contribution of *TGFBR1* to the CRC is less clear. (Valle *et al*, 2008) suggested that germline *TGFBR1* allele-specific expression could confer an increased CRC risk, although very recently, another study refuted this hypothesis (Seguí *et al*, 2011).

Another interesting gene located on region 9q22 is *GALNT12*, a member of the *N*-acetylgalactosaminyltransferase gene superfamily involved in protein glycosylation and highly expressed in the colon. Aberrant glycosylation is a known alteration that leads to the onset and progression of many cancers, including CRC. Recently, a study found germline mutations in the *GALNT12* gene in some CRC patients (Guda *et al*, 2009). We also included in our study eight *GALNT12* variants to further investigate whether the SNP variability of this gene could be involved in genetic susceptibility to CRC. However, we did not find any statistical significant association.

Although using a through and extensive two-phase case–control association study, we were not able to find any new susceptibility loci for CRC risk within these regions. However, some of the SNPs in region 3q22 showed interesting results in the joint analysis combining both cohorts (1361 CRC cases and 1342 controls), especially rs1444601 in *TOPBP1* and rs13088006 in *CDV3*, which maintained statistical significance with an unadjusted *P*-value in all phases. *TOPBP1* (topoisomerase DNA II-binding protein 1) represents a very interesting candidate for CRC genetic susceptibility as it contains multiple BRCT domains, the C-terminal portion of the *BRCA-1* gene, and it has a critical role in the control of DNA damage and replication checkpoint (Gong *et al*, 2010). On the other hand, *CDV3* (carnitine deficiency-associated gene expressed in ventricle 3), also known as *H41*, seems to be involved in cell proliferation and altered in gastric cancer (Oh *et al*, 2005). It is noteworthy that the *TOPBP1* and *CDV3* genes lie next to each other in 3q22 and rs1444601 and rs13088006 are only 34 kb apart. Therefore, it was interesting to know whether they co-segregated. Unfortunately, there were no available data for rs13088006 in HapMap. However, we used our own genotyping data in Haploview and found that they are not in the same haplotype block and segregate independently (*r*^2^=0.35).

When we checked our association results in two cohorts described in a previous GWAS (Tomlinson *et al*, 2007), none of these six variants were significantly associated with CRC risk. There could be differences in terms of the allele frequencies and linkage disequilibrium patterns between CORGI/VQ58 and EPICOLON data. This may explain the lack of replication of our suggestive hits in this external GWAS. In addition, the magnitude of the effect of a risk allele may differ between populations because of gene–gene or gene–environment interactions.

Finally, as limitations to our study, it should be commented that our cohort sample size is probably not large enough to be able to reach stronger conclusions for the analysed variants. However, our study (1416 CRC cases and 1424 controls for EPICOLON cohorts) had an estimated 80% power to detect an OR as low as 1.26 with an MAF of 0.30, 1.25 for a MAF down to 0.20 or 1.31 for a MAF down to 0.08, assuming a dominant model and *α*=0.05. It must be noted also that our conclusions were obtained in the EPICOLON cohorts and results were additionally corroborated in the CORGI and VQ58 cohorts, adding together 3721 CRC cases and 4970 controls. In addition, it should be commented that our results apply only for the analysed SNPs as we did not whatsoever comprehensively cover all possible low-penetrance genetic variants within the selected genes. Nevertheless, gene/SNP selection was biased to include genes with a plausible function in cancer and SNPs with a putative functional effect.

In conclusion, none of the 172 SNPs initially analysed in our study could be formally associated with CRC risk. However, variants in *TOPBP1* and *CDV3* showed interesting results and may have an effect on CRC risk. Despite our negative results, we consider additional case–control studies in larger CRC cohorts and meta-analysis could be useful to confirm or refute the role of *TOPBP1* and *CDV3* variants in CRC susceptibility.

## Figures and Tables

**Table 1:** SNPs with statistically significant results in EPICOLON stage 1 in the candidate-gene approach for regions 9q22 and 3q22

**SNP_ID**	**Gene**	**Location/relevance**	**Alleles**	**MAF cases**	**MAF controls**	**Test**	**GT counts cases**	**GT counts controls**	**OR (95% CI)**	***P*-value**
rs4149338	*ABCA1*	9q22/intron, triplex, mcons	C/T	0.241	0.285	Dom	201/280	248/252	0.73 (0.57–0.94)	0.0141
rs2515618	*ABCA1*	9q22/intron, triplex, mcons	G/A	0.255	0.278	Dom	213/287	250/260	0.77 (0.60–0.99)	0.0406
rs4149339	*ABCA1*	9q22/miss, ESE, mcons	C/T	0.217	0.278	Dom	183/300	247/257	0.63 (0.49–0.82)	0.00043
rs10991898	*AUH*	9q22/intron, triplex, mcons	C/T	0.095	0.119	Dom	87/394	117/386	0.73 (0.53–0.99)	0.0493^a^
rs589362	*C9ORF102*	9q22/3′UTR, ESE, mcons	C/G	0.320	0.358	Dom	257/223	306/198	0.75 (0.58–0.96)	0.0230
rs10123342	*NOL8*	9q22/promoter, mcons	T/G	0.343	0.394	Rec	52/433	86/407	0.57 (0.39–0.82)	0.0025
rs11466445	*TGFBR1*	9q22/3 AA deletion (GGCGGCGGC), mcons	3AA/-	0.084	0.109	Dom	77/406	105/399	0.72 (0.52–0.99)	0.0491^a^
rs12236219	*ZNF169*	9q22/miss, ESE, mcons	C/T	0.031	0.068	Allelic	30/940	69/939	0.43 (0.28–0.67)	0.00012^a^
rs2246945	*A4GNT*	3q22/miss, ESE, mcons	A/C	0.289	0.325	Rec	37/430	59/431	0.63 (0.40–0.97)	0.0340
rs2346747	*A4GNT*	3q22/promoter, mcons	G/A	0.317	0.352	Rec	46/434	69/417	0.64 (0.43–0.95)	0.0268
rs329387	*ARMC8*	3q22/intron, triplex, mcons	G/A	0.450	0.502	Dom	317/151	377/124	0.69 (0.52–0.91)	0.0095
rs939453	*ARMC8*	3q22/triplex, mcons	C/A	0.422	0.484	Allelic	404/554	484/516	0.78 (0.65–0.93)	0.0056
rs13088006	*CDV3*	3q22/intron, triplex, mcons	T/C	0.232	0.256	Rec	22/458	40/448	0.54 (0.31–0.92)	0.0217
rs1673607	*CEP70*	3q22/miss, ESE, ESS, mcons	A/G	0.472	0.501	Rec	108/388	140/367	0.73 (0.55–0.97)	0.0321
rs811322	*FAIM*	3q22/synon, ESE	A/G	0.456	0.485	Rec	98/389	128/373	0.73 (0.54–0.99)	0.0423
rs108858	*IL20RB*	3q22/3′UTR, ESE	A/G	0.433	0.475	Dom	313/161	362/136	0.73 (0.56–0.96)	0.0243
rs3738000	*NEK11*	3q22/miss, mcons	T/A	0.273	0.301	Dom	218/262	261/239	0.76 (0.59–0.98)	0.0337
rs10934954	*PIK3R4*	3q22/synon, ESS	C/T	0.242	0.204	Rec	33/446	19/481	1.87 (1.05–3.34)	0.0311
rs2071387	*RBP1*	3q22/intron, mcons	T/C	0.170	0.177	Rec	8/475	22/478	0.37 (0.16–0.83)	0.0124
rs1131597	*SLCO2A1*	3q22/3′UTR, mcons	G/A	0.303	0.379	Allelic	289/665	380/622	0.71 (0.59–0.86)	0.00038
rs1444601	*TOPBP1*	3q22/synon, mcons	A/G	0.238	0.281	Allelic	231/739	282/720	0.80 (0.65–0.98)	0.0284

Abbreviations: AA=amino acid; CI=confidence interval; Dom=dominant; ESE=exonic splicing enhancer; ESS=exonic splicing silencer; GT=genotype; MAF=minor allele frequency; mcons=conserved in mouse; miss=missense; Rec=recessive; SNP_ID=SNP identification; synon=synonymous; intron=intronic; UTR=untranslated region.

Results are shown according to the best fitting-model.

a If one of the genotypes had a frequency <5, then Fisher's exact test was used.

**Table 2:** Summary results for the six most significant SNPs in all EPICOLON phases

			**Discovery**		**Replication**		**Overall**	
**SNP**	**Gene**	**Test**	**OR (95% CI)**	***P*-value**	**OR (95% CI)**	***P*-value**	**OR (95% CI)**	***P*-value**
rs1444601	*TOPBP1*	Geno	0.77 (0.59–1.00)		0.93 (0.76–1.14)		0.88 (0.75–1.03)	
			0.69 (0.42–1.13)	0.087	0.64 (0.43–0.97)	0.097	0.65 (0.48–0.89)	**0.015**
		Allelic	0.80 (0.65–0.98)	**0.028**	0.86 (0.74–1.01)	0.070	0.84(0.75–0.95)	**0.0054**
		Dom	0.76 (0.59–0.97)	**0.030**	0.88 (0.73–1.07)	>0.1	0.84 (0.72–0.98)	**0.023**
		Rec	0.77 (0.47–1.24)	0.279	0.66 (0.45–0.99)	**0.041**	0.69 (0.51–0.94)	**0.016**
								
rs13088006	*CDV3*	Geno	1.05 (0.80–0.37)		0.83 (0.68–1.03)		0.92 (0.78–1.08)	
			0.55 (0.32–0.95)	0.067	0.73 (0.46–1.16)	>0.1	0.66 (0.47–0.92)	**0.043**
		Allelic	0.88 (0.71–1.08)	0.222	0.84 (0.71–0.99)	**0.042**	0.87 (0.76–0.98)	**0.025**
		Dom	0.96 (0.74–1.24)	0.715	0.82 (0.67–0.99)	**0.049**	0.88 (0.75–1.02)	0.098
		Rec	0.54 (0.31–0.92)	**0.022**	0.78 (0.49–1.23)	>0.1	0.98 (0.48–0.95)	**0.022**
								
rs939453	*ARMC8*	Geno	0.68 (0.51–0.90)		1.10 (0.88–1.38)		1.10 (0.71–1.01)	
			0.62 (0.43–0.89)	**0.017**	1.07 (0.81–1.41)	>0.1	0.79 (0.64–0.98)	0.068
		Allelic	0.78 (0.65–0.93)	**0.0056**	0.96 (0.84–1.1)	>0.1	0.89 (0.80–0.99)	**0.025**
		Dom	1.47 (1.11–1.93)	**0.0062**	0.91 (0.74–1.13)	>0.1	0.83 (0.71–0.98)	0.27
		Rec	0.77 (0.56–1.04)	0.091	0.99 (0.78–1.25)	>0.1	0.88 (0.73–1.05)	0.163
								
rs1131597	*SLCO2A1*	Geno	0.77 (0.59–1.01)		0.97 (0.79–1.19)		0.97 (0.76–1.04)	
			0.47 (0.31–0.72)	**0.0015**	1.07 (0.79–1.47)	>0.1	0.80 (0.63–1.02)	>0.1
		Allelic	0.71 (0.59–0.86)	**0.00038**	1.02 (0.88–1.17)	>0.1	0.89 (0.80–0.99)	**0.043**
		Dom	0.70 (0.54–0.90)	**0.005**	0.99 (0.82–1.20)	>0.1	0.88 (0.75–1.01)	0.068
		Rec	0.54 (0.36–0.81)	**0.0026**	1.09 (0.81–1.46)	>0.1	0.85 (0.67–1.07)	0.164
								
rs10934954	*PIK3R4*	Geno	1.13 (0.86–1.47)		0.93 (0.75–1.15)		1.01 (0.85–1.18)	
			1.95 (1.09–3.51)	0.068	1.25 (0.79–1.98)	>0.1	1.50 (1.05–2.13)	0.081
		Allelic	1.25 (1.01–1.54)	**0.042**	1.01 (0.87–1.15)	>0.1	1.10 (0.97–1.25)	>0.1
		Dom	1.21 (0.94–1.56)	0.146	0.97 (0.79–1.18)	>0.1	1.06 (0.91–0.24)	0.450
		Rec	1.87 (1.05–3.34)	**0.031**	1.28 (0.82–2.02)	>0.1	1.49 (1.05–2.12)	**0.025**
								
rs2071387	*RBP1*	Geno	1.17 (0.89–1.55)		1.02 (0.82–1.26)		1.04 (0.88–1.23)	
			0.38 (0.17–0.87)	**0.023**	1.40 (0.81–2.43)	>0.1	0.57 (0.36–0.90)	**0.034**
		Allelic	0.95 (0.75–1.20)	0.672	0.93 (0.78–1.11)	>0.1	0.93 (0.81–1.07)	>0.1
		Dom	1.06 (0.81–1.39)	0.662	0.95 (0.77–1.17)	>0.1	0.98 (0.84–1.15)	>0.1
		Rec	0.37 (0.16–0.83)	**0.012**	0.72 (0.42–1.24)	>0.1	0.56 (0.36–0.88)	**0.010**

Abbreviations: CI=confidence interval; Dom=dominant; Geno=genotypic; OR=odds ratio; Rec=recessive; SNP=single-nucleotide polymorphism.

ORs and unadjusted *P*-values are shown for discovery, replication and overall phases for each of them. Significant *P*-values are depicted in bold.

**Table 3:** Association results for six selected SNPs were evaluated in two cohorts (CORGI and VQ58) in an external GWAS, either by checking the original variant or a proxy SNP highly correlated with it (*r*^2^>0.7).

	**Present in I5?**	**Present in I3?**	**Proxy**	***r*^2^ and D′**		***P* (CORGI)**	***P* (VQ58)**
rs1444601	Yes	Yes	—			0.2653	0.7039
rs13088006	No	No	rs6769437	0.818	1.000	0.5816	0.4101
rs939453	No	No	rs7631734	0.869	0.964	0.7984	0.2922
rs1131597	No	No	rs7616492	0.762	1.000	0.1273	0.9246
rs10934954	No	No	rs2200368	1.000	1.000	0.6710	0.0899
rs2071387	Yes	Yes	—			0.7243	0.8671

Abbreviations: GWAS=genome-wide association study; SNP=single-nucleotide polymorphism; I5=Illumina HumanHap550; I3=Illumina HumanHap300 (Illumina, San Diego, CA, USA).

## Supplementary Material

Supplementary Notes
