# Supplementary material for: A two-phase case–control study for colorectal cancer genetic susceptibility: candidate genes from chromosomal regions 9q22 and 3q22
Source: Br J Cancer. 2011 Aug 2;105(6):870–5. doi: 10.1038/bjc.2011.296 (PMC3171011; doi:10.1038/bjc.2011.296)
Supplement: Supplementary Table 1 [file bjc2011296x2.doc]

**Supplementary Table 1.** Description of selected genes and SNPs from linkage region on chromosome 9.

| Gene | **Reference position*** | **Gene description** | **GO terms** | **SNPs** |
| --- | --- | --- | --- | --- |
| *LOC401537* | 90939296-90940325 | Similar to Laminin receptor 1 | Cell adhesion,signal transduction | rs3750493 |
| *S1PR3* | 91606362-91619925 | Sphingosine-1-phosphate receptor 3 | G-protein coupled receptor activity, lipid binding | rs3934594  rs1556385 |
| *CKS2* | 91926113-91931618 | CDC28 protein kinase regulatory subunit 2 | Cell cycle control | rs1328304 |
| *GADD45G* | 92219927-92221470 | Growth arrest and DNA-damage-inducible, gamma | DNA repair, apoptosis, cell differentiation | rs3138488 |
| *DIRAS2* | 93372114-93405108 | DIRAS family, GTP-binding RAS-like 2 | Signal transduction | rs690111 |
| *SYK* | 93564012-93660842 | Spleen tyrosine kinase | Cell proliferation | rs2306041  rs1049164 |
| *AUH* | 93976097-94124206 | AU RNA binding protein/enoyl-CoA hydratase | mRNA catabolic process | rs10991898 |
| *NOL8* | 95059640-95087876 | Nucleolar protein 8 | Nucleotide binding, positive regulation of cell growth | rs9409469  rs10123342 |
| *ZNF484* | 95608351-95640290 | Zinc finger protein 484 | Transcriptional regulation | rs1040978 |
| *SUSD3* | 95820989-95847415 | Sushi domain containing 3 | Integral to memebrane, sushi domain | rs4077812 |
| *NINJ1* | 95883771-9896570 | Ninjurin 1 | Cell adhesion, tissue regeneration | rs2275848 |
| *WNK2* | 95947212-96082854 | Lysine deficient protein kinase 2 | Signal transduction | rs10761203  rs3001450 |
| *PHF2* | 96338909-96441869 | PHD finger protein 2 | Protein binding | rs8690 |
| *PTPDC1* | 96793076-9672136 | Protein tyrosine phosphatase domain containing 1 | Signal transduction | rs10821295 |
| *ZNF169* | 97021578-97065291 | Zinc finger protein 169 | Transcriptional regulation | rs1536690  rs12236219 |
| *FANCC* | 97861336-98079991 | Fanconi anemia, complementation group C | DNA repair | rs4647558  rs356663 |
| *PTCH1* | 98205264-98279247 | Patched 1 | Regulation of smoothened signaling pathway | rs357564  rs2066836 |
| *C9ORF102* | 98637900-98731122 | Chromosome 9 open reading frame 102 | DNA repair, putative recombination helicase RAD26L | rs16910370  rs589362 |
| *ZNF367* | 99148223-99180669 | Zinc finger protein 367 | Transcriptional regulation | rs7861768 |
| *HABP4* | 99212414-99253618 | Hyaluronan binding protein 4 | Transcriptional regulation | rs7030316  rs6477493 |
| *CDC14B* | 99262395-99382112 | CDC14 cell division cycle 14 homolog B | DNA repair, G2/M transition DNA damage checkpoint | rs10820745  rs7027967 |
| *LOC441455* | 99488103-99489749 | Similar tp Makorin 1 | DNA binding | rs16911482 |
| *ZNF510* | 99518147-99540328 | Zinc finger protein 510 | Transcriptional regulation | rs2289651  rs2289649 |
| *ZNF782* | 99579273-99616389 | Zinc finger protein 782 | Transcriptional regulation | rs7848710 |
| *CTSL2* | 99794937-99801539 | Cathepsin L2 | Proteolysis | rs15394 |
| *TDRD7* | 100174302-100258407 | Tudor domain containing 7 | Nucleid acid binding | rs2045732 |
| *NCBP1* | 100395705-100436030 | Nuclear cap binding protein subunit 1 | RNA silencing | rs2773350  rs2297155 |
| *XPA* | 100437191-100459691 | Xeroderma pigmentosum, complementation group A | Nucleotid-excison repair, DNA damage removal | rs1800975 |
| *ANP32B* | 100745489-100778225 | Acidic (leucine-rich) nuclear phosphoprotein 32 family, member B | cell cycle progression factor and cell survival factor | rs987142 |
| CORO2A | 100883257-100954956 | coronin, actin binding protein, 2A | Signal transduction, transcriptional repressor complex | rs774123  rs3780457 |
| *TBC1D2* | 100961280-101018003 | TBC1 domain family, member 2 | Regulation of Rab GTPase activity | rs3780453  rs12348328  rs879369  rs879368  rs7853442  rs7047722 |
| *GALNT12* | 101569981-101612359 | UDP-N-acetyl-alpha-D-galactosamine:polypeptide N-acetylgalactosaminyltransferase 12 (GalNAc-T12) | Plays an important role in the initial step of mucin-type oligosaccharide biosynthesis in digestive organs. | rs1543506  rs1555519  rs10819320  rs2273846  rs7043234  rs999471  rs2295925  rs10987898 |
| *TGFBR1* | 101867412-101916474 | Transforming growth factor, beta receptor 1 | Activation of MAPK activity | rs11466445  rs7031302  rs2026811  rs10988714  rs334356  rs868  rs334348 |
| *TMEFF1* | 103235717-103339914 | Transmembrane protein with EGF-like and two follistatin-like domains 1 | Tumour suprressor? | rs2026423  rs2800284 |
| *BAAT* | 104122699-104147287 | Bile acid CoA: amino acid N-acyltransferase (glycine N-choloyltransferase) | Involved in bile acid metabolism | rs7043029  rs2229594  rs12350797  rs1572983  rs3824471  rs1891218 |
| *ZNF189* | 104161163-104172942 | Zinc finger protein 189 | Transcriptional regulation | rs1929491  rs546577 |
| *RNF20* | 104296133-104325626 | Ring finger protein 20 | Histone H2B ubiquitination | rs7872953  rs4743471 |
| *SMC2* | 106856541-106903700 | Structural maintenance of chromosomes 2 | Cell division control | rs1360176  rs15532 |
| *ABCA1* | 107543283-107690436 | ATP-binding cassette, sub-family A, member 1 | Cholesterol transporter; signal transduction | rs4149339  rs4149338  rs2230808  rs4149313  rs2230806  rs2254819  rs4149265  rs2515618  rs2487049 |
| *ZNF462* | 109625378-109773807 | Zinc finger protein 462 | Transcriptional regulation | rs10978675  rs17723637  rs3814541  rs12003150  rs10217192  rs1805329 |
| *RAD23B* | 110045544-110094470 | RAD23 homolog B | Nucleotid-excison repair, DNA damage recognition | rs1805329 |

*According to NCBI build 37.2 available at <http://www.ncbi.nlm.nih.gov/sites/gene>
