# Supplementary material for: A two-phase case–control study for colorectal cancer genetic susceptibility: candidate genes from chromosomal regions 9q22 and 3q22
Source: Br J Cancer. 2011 Aug 2;105(6):870–5. doi: 10.1038/bjc.2011.296 (PMC3171011; doi:10.1038/bjc.2011.296)
Supplement: Supplementary Table 2 [file bjc2011296x3.doc]

**Supplementary table 2.** Description of selected genes and SNPs from linkage region on chromosome 3.

| Gene | **Mapping** | **Gene description** | **GO terms** | **SNPs** |
| --- | --- | --- | --- | --- |
| *PLXND1* | 129274056-129325582 | Plexin D1 | Signal transduction | rs1052883  rs2625973 |
| *TRH* | 129693114-129696778 | Thyrotropin-releasing hormone | Signal transduction | rs2670899  rs5658 |
| *LOC646300* | 129931663-129992649 | Similar to alpha 3 type VI collagen isoform precursor | Cell adhesion | rs6777871  rs9849611 |
| *COL29A1* | 130064359-130203688 | collagen, type VI, alpha 5 | Cell adhesion | rs819085  rs322117 |
| *COL6A6* | 130279178-130395888 | collagen, type VI, alpha 6 | Cell adhesion | rs9830253  rs16829695 |
| *PIK3R4* | 130397778-130465696 | Phosphoinositide-3-kinase, regulatory subunit 4 | Fibrobalst growth factor receptor signaling pathway | rs10934954  rs2293179 |
| *ASTE1* | 130732721-130745646 | Asteroid homolog 1 | DNA repair | rs218502  rs13065390 |
| *NEK11* | 130745694-131069309 | NIMA (never in mitosis gene a)- related kinase 11 | Cell cycle control | rs3738000  rs16836266 |
| *NUDT16* | 131080689-131083966 | Nudix (nucleoside diphosphate linked moiety X)-type motif 16 | RNA binding, RNA decay | rs3749390 |
| *CPNE4* | 131253572-131753844 | Copine IV | Phospholipid-binding protein | rs11915192  rs17398428 |
| *ACPP* | 132036211-132087146 | Acid phosphatase, prostate | Acid phosphatase activity, prostate tumour marker | rs3844501  rs2198765 |
| *DNAJC13* | 132136553-132257876 | DnaJ (Hsp40) homolog, subfamily C, member 13 | Protein binding, EGFR regulation | rs3762672 |
| *ACAD11* | 132276982-132378975 | Acyl-CoA dehydrogenase family, member 11 | Oxidation-reduction process | rs2305627  rs10935023 |
| *CCRL1* | 132316094-132321382 | Chemokine (C-C motif) receptor-like 1 | Chemotaxis | rs7626622 |
| *UBA5* | 132373290-132396944 | Ubiquitin-like modifier activating enzyme 5 | Protein degradation | rs2168435  rs1058331 |
| *CDV3* | 133292434-133309118 | CDV3 homolog (mouse) | Cell proliferation | rs13088006 |
| *TOPBP1* | 133319449-133380737 | Topoisomerase (DNA) II binding protein 1 | DNA repair | rs1444601  rs17301766 |
| *TF* | 133464977-133497850 | Transferrin | Cellular iron ion homeostasis | rs1799852  rs1049296 |
| *SRPRB* | 133502877-133540336 | Signal recognition particle receptor, B subunit | GTP binding | rs9853615 |
| *RAB6B* | 133543079-133614691 | Member RAS oncogene family | Signal transduction | rs1043147  rs6765093 |
| *SLCO2A1* | 133651540-133748920 | Solute carrier organic anion transporter family, member 2A1 | Sodium-independent organic anion transport | rs1131597  rs34550074 |
| *RYK* | 133875978-133969586 | Receptor-like tyrosine kinase | Potential growth factor receptor protein tyrosine kinase. | rs7631521  rs1131262 |
| *AMOTL2* | 134074190-134093406 | Angiomotin like 2 | Angiogenesis | rs9450  rs1353776 |
| *ANAPC13* | 134196546-134204865 | Anaphase promoting complex subunit 13 | Cell cycle control | rs1863913 |
| *CEP63* | 134204575-134293855 | Centrosomal protein 63kDa | Cell cycle control | rs6772896  rs6804770 |
| *EPHB1* | 134514099-134979309 | EPH receptor B1 | Signal transduction | rs7644369  rs3182239 |
| *PPP2R3A* | 135684515-135866752 | Protein phosphatase 2, regulatory subunit B'', alpha | Cell growth control, Wnt pathway | rs9883808  rs6779903 |
| *MSL2* | 135867760-135914688 | Male-specific lethal 2 homolog (Drosophila) | Histone H4-K16 acetylation | rs7372313  rs9845457 |
| *STAG1* | 136055999-136471245 | Stromal antigen 1 | Chromatid cohesion | rs900818 |
| NCK1 | 136581050-136667968 | Adaptor protein 1 | Positive regulation of T cell proliferation | rs9836807  rs10804645 |
| *IL20RB* | 136676707-136729920 | Interleukin 20 receptor beta | Blood coagulation | rs7648198  rs108858 |
| *CLDN18* | 137717658-137752494 | Claudin 18 | Calcium-independent cell-cell adhesion | rs17204075  rs9858250 |
| *A4GNT* | 137842560-137851229 | Alpha-1,4-N-acetylglucosaminyltransferase | Protein O-linked glycosilation | rs2246945  rs2346747 |
| *DBR1* | 137879830-137893791 | Debranching enzyme homolog 1 (S. cerevisiae) | mRNA processing | rs2622737  rs329379 |
| *ARMC8* | 137906148-138016219 | Armadillo repeat containing 8 | Protein interaction, same protein domain in APC | rs329387  rs939453 |
| *MRAS* | 138066625-138124377 | Muscle RAS oncogene homolog | Ras protein signal transduction | rs3755751  rs2279241 |
| *CEP70* | 138213186-138313129 | Centrosomal protein 70kDa | G2/M transition of mitotic cell cycle | rs9049  rs1673607 |
| *FAIM* | 138327542-138352218 | Fas apoptotic inhibitory molecule | Negative regulation of apoptosis | rs811322  rs641320 |
| *PIK3CB* | 138374231-138478185 | Phosphoinositide-3-kinase, catalytic, beta polypeptide | Signal transduction | rs12493155  rs361072 |
| *RBP2* | 139171726-139195352 | Retinol binding protein 2, cellular | Intracellular transport of retinol | rs3772877  rs17336869 |
| *RBP1* | 139236276-139258671 | Retinol binding protein 1, cellular | Intracellular transport of retinol | rs6797111  rs2071387 |
| *NMNAT3* | 139279033-139396840 | Nicotinamide nucleotide adenylyltransferase 3 | Pyridine nucleotid biosynthesis | rs3922941 |
| *CLSTN2* | 139654027-140286919 | Calsyntenin 2 | Cell adhesion | rs7632885  rs349537 |

*According to NCBI build 37.2 available at <http://www.ncbi.nlm.nih.gov/sites/gene>
